# PGT-Net: A Physics-Guided Transformer–CNN Hybrid Network for Low-Light Image Enhancement and Object Detection in Traffic Scenes

**DOI:** 10.3390/jimaging12050191

**Published:** 2026-04-28

**Authors:** Bin Chen, Jian Qiao, Baowei Li, Shipeng Liu, Wei She

**Affiliations:** 1 School of Computer and Artificial Intelligence, Zhengzhou University, Zhengzhou 450002, China; cbcqx123456@163.com; 2 XJ Electric Co., Ltd., Xuchang 461000, China; baowei_li228@163.com; 3 State Key Laboratory of Alternate Electrical Power System with Renewable Energy Sources, North China Electric Power University (Baoding), Baoding 071003, China; qiaojian@ncepu.edu.cn; 4 School of Cyber Science and Engineering, Zhengzhou University, Zhengzhou 450002, China; liushipeng@gs.zzu.edu.cn; 5 State Key Laboratory of Target Vulnerability Assessment, Luoyang 471023, China

**Keywords:** low-light enhancement, object detection, physics-guided learning, transformer, CNN, autonomous driving

## Abstract

In autonomous driving and intelligent transportation systems, the degradation of image quality under low-light conditions severely impacts the reliability of subsequent object detection. Existing methods predominantly employ data-driven deep learning models for image enhancement, often lacking physical interpretability and struggling to maintain robustness in complex lighting-varying traffic scenarios. To address this, this paper proposes a Physically Guided Transformer–CNN Hybrid Network (Physically Guided Transformer–CNN Hybrid Network, PGT-Net) for end-to-end joint optimization of low-light enhancement and object detection. PGT-Net innovatively integrates the atmospheric scattering physical model with deep learning architecture: first, a learnable physical guidance branch estimates the scene’s atmospheric illumination map and transmittance map, providing explicit physical priors for the network; second, a dual-branch enhancement backbone is designed, where the local CNN branch (based on an improved UNet) restores fine textures, while the Global Transformer Branch (based on Swin Transformer) models long-range dependencies to correct global uneven illumination, with features adaptively combined via a Physical Fusion Module to ensure enhancement results align with physical laws while retaining rich visual features; finally, the enhanced images are directly fed into a lightweight detection head (e.g., YOLOv7) for joint training and optimization. Comprehensive experiments on public datasets (ExDark, BDD100K-night, etc.) demonstrate that PGT-Net significantly outperforms mainstream methods (e.g., RetinexNet, KinD, Zero-DCE) in both low-light image enhancement quality (PSNR/SSIM) and object detection accuracy (mAP), while maintaining high inference efficiency. This research offers an interpretable, high-performance solution for visual perception tasks under adverse lighting conditions, holding strong theoretical significance and practical value.

## 1. Introduction

### 1.1. Research Background and Significance

With the rapid advancement of artificial intelligence, autonomous driving and intelligent transportation systems are transitioning from concept to reality [1]. In these systems, computer vision-based environmental perception plays a critical role [2]. However, real-world road environments are complex, particularly under low-light conditions such as nighttime, tunnels, dawn, and dusk, which severely degrade visual perception performance. Low-light images typically suffer from low contrast, detail loss, color distortion, and severe noise [3], leading to a significant decline in the accuracy of downstream tasks like object detection, recognition, and tracking. According to the World Health Organization, approximately 40% of global traffic accidents occur at night, while nighttime traffic accounts for only 25% of total daily traffic [4], highlighting the serious challenges to driving safety in low-light environments.

Traditional solutions fall into two categories: hardware improvements (e.g., high-sensitivity sensors, infrared imaging [5]) and software-based enhancement algorithms. Hardware upgrades are often costly and face physical constraints, whereas software approaches offer greater flexibility and cost-effectiveness. In recent years, deep learning has significantly advanced low-light image enhancement [6]. However, most existing deep learning methods are purely data-driven, lacking explicit modeling of the physical degradation process. This often leads to limited generalization in complex traffic scenes, with enhancement results suffering from artifacts, over-enhancement, or under-enhancement [7], ultimately degrading downstream detection performance.

Therefore, exploring a solution that combines physical priors with deep learning, balancing image enhancement quality and object detection performance, holds important theoretical and practical value. This study aims to fill this gap and provide reliable technical support for safe autonomous driving under harsh lighting conditions.

### 1.2. Current Research Status at Home and Abroad

#### 1.2.1. Low-Light Image Enhancement Method

Low-light image enhancement methods can be divided into traditional and deep learning approaches.

**Traditional methods** include histogram equalization (HE) and its variants, which enhance contrast by redistributing pixel intensities but often amplify noise and cause local over-enhancement [8]; Retinex theory-based methods [9], which decompose an image into reflection and illumination components—representative approaches include SSR, MSR, and MSRCR—which effectively improve brightness and contrast but are sensitive to parameters and prone to halo artifacts in complex scenes; and dehazing model-based methods, which treat low-light images as the inverse of foggy images and enhance them using atmospheric scattering models, such as LIME [10] and MF [11], offering strong physical interpretability but requiring accurate illumination map estimation.

**Deep learning methods** have become mainstream and can be categorized into:

**CNN-based supervised learning:** LLNet [12], RetinexNet [6], and KinD [7] combine Retinex decomposition with deep learning. RetinexNet uses two subnetworks to estimate illumination and reflection components separately, while KinD introduces a lighting adjustment network for multi-stage refinement. These methods perform well on paired datasets but rely heavily on data quality and lack global modeling capability.

**Unsupervised/self-supervised methods:** EnlightenGAN [13] and Zero-DCE [14] use adversarial training or curve estimation to train without paired data. Zero-DCE learns pixel-level high-order curves to adjust dynamic range but may overlook spatial context.

**Transformer-based methods:** Uformer [15] and Restormer [16] introduce Transformer structures into image restoration, capturing long-range dependencies via self-attention. However, pure Transformer models are weaker at local detail recovery and have higher computational complexity.

#### 1.2.2. Object Detection Under Low-Light Conditions

Object detection under low-light faces the dual challenges of poor image quality and limited annotated data. Existing methods are mainly divided into two-stage and end-to-end joint optimization strategies.

The two-stage strategy (“enhancement first, detection later”) offers high flexibility but lacks task-specific optimization; the enhancement stage may not benefit the detector and can even introduce harmful artifacts [17]. In contrast, end-to-end joint optimization uses multi-task learning to co-train enhancement and detection modules, as exemplified by JLOD [18]. While this approach can learn detection-friendly features, it may suffer from unstable training and potential visual quality degradation.

Recent advances can be categorized into three main directions. The first focuses on learnable image processing pipelines, such as IA-YOLO [19], which incorporates a differentiable image processing module (DIP) to adaptively enhance low-light images before detection. This approach demonstrates the benefits of task-specific pre-processing but treats enhancement and detection as separate, sequentially connected modules. The second direction explores unsupervised or self-supervised feature learning for detection, exemplified by Zero-Det [14] and DSLR [20], which aim to learn detection-friendly representations directly from unpaired low-light images. While these methods reduce reliance on paired training data, they often lack explicit physical interpretability. The third direction investigates joint optimization frameworks, where the enhancement and detection modules are trained together. Approaches like JLOD [21] and the detection head in IA-YOLO show promise, but they typically employ simple feature concatenation or shared loss functions without deeply integrating physical priors. Despite these advances, most methods remain limited to CNN architectures, which struggle to model long-range dependencies essential for correcting global illumination unevenness in complex traffic scenes [22]. Furthermore, the integration of physical priors into the learning process remains shallow, limiting interpretability and generalization.

#### 1.2.3. Research on the Integration of Physical Models and Deep Learning

Integrating physical priors into deep learning to enhance interpretability and generalization is an important research direction, with explorations in dehazing, deraining, and super-resolution [21,22]. For dehazing, many works use atmospheric scattering models as network design bases, estimating transmittance and atmospheric light via subnetworks [22]. In low-light enhancement, RUAS [23] proposes a deep unfolding architecture compatible with the Retinex model. However, most existing fusion methods treat physical models as fixed constraints or simple initializations, failing to achieve deep and adaptive fusion with data-driven features. Application research in complex traffic scenes remains insufficient.

In contrast, the proposed PGT-Net introduces a learnable physical guidance branch that estimates spatially varying atmospheric illumination and transmittance maps, enabling deep, adaptive fusion with data-driven features. Application research in complex traffic scenes remains insufficient, and our work specifically addresses this gap through a unified framework that integrates physical priors, local detail restoration, and global context modeling.

Beyond traditional vision tasks, physics-guided hybrid paradigms are gaining momentum across imaging domains. For instance, recent work in medical imaging has explored quantum neural networks integrated with physics-based priors [24], underscoring the value of combining domain-specific physical knowledge with advanced neural architectures.

#### 1.2.4. Summary of Existing Problems

By systematically reviewing existing research, the following key issues that urgently need to be addressed can be summarized:

**Insufficient deep integration of physical priors:** Existing methods either rely on purely data-driven learning or treat physical models as fixed pre-processing steps. Recent approaches like IA-YOLO [19] and JLOD [21] use enhancement as a pre-processing module but fail to embed physical laws into the network in a deep, learnable manner. This limits the model’s interpretability and generalization, especially under unseen lighting conditions.

**Limited capability for joint global–local context modeling:** While CNN-based methods (e.g., KinD [7], Zero-Det [16]) excel at local detail recovery, they are inherently constrained in capturing long-range dependencies necessary for correcting global illumination issues like glare or large shadows. Conversely, pure Transformer-based methods (e.g., Uformer [15]) are computationally expensive and may sacrifice local detail fidelity. An efficient and adaptive complementarity between local and global features remains an open challenge.

**Lack of task-driven joint optimization:** Most two-stage “enhance-then-detect” strategies [19,21] suffer from a task gap, as the enhancement module is not explicitly optimized for downstream detection performance. Existing end-to-end methods often use simple feature sharing or multi-task losses without a deep, physically guided interaction between the two tasks. This prevents the enhancement process from learning detection-friendly feature representations.

**Underutilization of traffic scene structure:** Generic enhancement methods do not fully exploit structured priors inherent to traffic scenes, such as the spatial distribution of light sources (e.g., headlights, street lamps) or typical target scales, leading to suboptimal robustness in complex dynamic scenes.

To clearly illustrate the limitations of existing methods, Table 1 compares different technical routes.

### 1.3. The Main Contribution of This Article

In response to the above issues, this paper proposes PGT-Net (Physically Guided Transformer–CNN Hybrid Network), with the following main contributions:

**Learnable physics-guided decomposition mechanism.** Unlike existing methods that treat physical priors as fixed constraints or simple initialization, this paper proposes a learnable Physical Guided Branch (PGB) that can explicitly estimate the spatially varying atmospheric illumination map A and transmittance map t. This provides strong physical priors, improves generalization ability in the absence of illumination, and avoids errors caused by manual estimation.

**Efficient dual-branch enhanced backbone network.** This article proposes a parallel architecture that integrates local CNN branches (improved UNet++) and Global Transformer Branches (lightweight Swin Transformer). Among them, the CNN branch focuses on restoring fine details, while the Transformer branch is used to model long-range dependencies to correct global uneven lighting. These two are adaptively fused through the Physical Fusion Module (PFM), achieving deep complementarity at the pixel level, feature level, and physical law level.

**End-to-end enhancement and detection joint optimization.** This article constructs a fully end-to-end trainable framework that deeply integrates a physics-guided enhanced backbone network with a lightweight detection head (YOLOv7-tiny). By introducing a multi-task loss function that integrates physical consistency, image quality, perceptual loss, and detection loss, we ensure enhanced direct assistance detection performance while maintaining visual fidelity.

**State-of-the-art performance on traffic datasets**. Comprehensive experiments on challenging public low-light traffic datasets (ExDark, BDD100K-night, SID) demonstrate that PGT-Net significantly outperforms existing methods in both image enhancement quality (PSNR, SSIM, LPIPS) and object detection accuracy (mAP), while maintaining real-time inference speed suitable for autonomous driving applications.

## 2. Method Introduction

### 2.1. Physical Model for Low-Light Image Enhancement

#### 2.1.1. Atmospheric Scattering Model

The atmospheric scattering model is commonly used to describe image degradation under hazy conditions [25]. Research has found an inverse relationship between low-light images and foggy images [10], making this model applicable to low-light enhancement. The mathematical model is as follows:

(1) I(x)=J(x)⋅t(x)+A⋅(1−t(x))

where I(x) is the pixel value of the observed low-light image at position x,J(x) is the clear scene irradiance to be restored, A is the global atmospheric illumination (usually assumed to be constant or slowly changing), $t(x)=e^{-\beta \cdot d(x)}$ is the scene transmittance, β is the atmospheric scattering coefficient, and d(x) is the scene depth.

The physical meaning of this model is clear: the light intensity received by the camera consists of two parts—the directly attenuated object-reflected light J(x)⋅t(x) and the background light A⋅(1−t(x)) formed by atmospheric light scattering.

The physical meaning of transmittance t(x) is the degree of attenuation of light before it reaches the camera, which can be further expressed as follows:

(2) $$t(x)=e^{-\beta (\lambda )\cdot d(x)}$$

where β(λ) represents the wavelength-dependent scattering coefficient. In single-channel simplified models, wavelength dependence is usually ignored.

The physical meaning is clear: the camera receives both directly attenuated object-reflected light and background light formed by atmospheric scattering. Equation (1) can be rearranged to solve for the clear image:

(3) $$J(x)=\frac{I(x)-A\cdot (1-t(x))}{t(x)}$$

In practice, to maintain numerical stability, a small constant $\varepsilon =10^{-6}$ is introduced:

(4) $$J(x)=\frac{I(x)-A}{\max(t(x),\varepsilon )}+A$$

Low-light images typically have low I(x) values, making the J(x)⋅t(x) term weak and the A⋅(1−t(x)) term dominant. The goal of enhancement is to accurately estimate A and t(x) to recover J(x). This model provides a clear physical explanation: A represents ambient lighting, and t(x) describes light attenuation.

**Limitations of traditional methods**: In traditional dehazing or low-light enhancement, A is often assumed to be a global constant (e.g., the brightest pixel), and t(x) is estimated via dark channel priors or heuristics. However, in complex traffic scenes, strong light sources cause spatial variation in A, and complex depth distributions limit heuristic estimation accuracy.

**Core idea of learnable physics guidance**: To overcome these limitations, we embed the atmospheric scattering model into a neural network in a learnable manner. Instead of treating A and t(x) as fixed priors, we design a lightweight fully convolutional network (PGB) to predict spatially varying A(x) and t(x) end-to-end from the observed image.

**Limitations and scope of the atmospheric scattering model:** Despite its wide applicability, the atmospheric scattering model has inherent limitations when applied to low-light enhancement. First, the model assumes that scene radiance is attenuated by a homogeneous scattering medium, which may not hold in complex traffic scenes with dynamic light sources (e.g., headlights, street lamps) that introduce localized illumination variations [10]. Second, the model does not explicitly account for sensor noise, which is often amplified in low-light conditions and can lead to unnatural artifacts after enhancement [7]. Third, the transmittance map t is depth-dependent, but the model assumes a simplified relationship that may not capture fine-grained depth variations in cluttered traffic scenes. Recent studies have attempted to address these limitations by combining the atmospheric scattering model with noise-aware priors [16] or learning-based depth estimation [22]. In this work, we mitigate these limitations by learning spatially varying A and t in a data-driven manner, allowing the network to adapt to complex lighting distributions, while the physical consistency loss (Section 4.1) provides regularization to preserve physically plausible enhancement.

#### 2.1.2. Retinex Theory

Retinex theory [9] decomposes an image S(x) into the product of illumination L(x) and reflection R(x):

(5) S(x)=L(x)⋅R(x)

where L(x) represents the light shining on the surface of the object, which changes slowly; R(x) represents the reflection characteristics of an object’s surface, including details and color information of the image, with drastic changes. The goal of low-light enhancement is to estimate L from S and then obtain the reflection component by R=S/L. Usually, L is corrected (such as adjusting the gamma value) and then multiplied with R to obtain the enhanced result. 

Taking logarithms converts multiplication to addition:

(6) logS(x)=logL(x)+logR(x)

L(x) is typically low-frequency and smooth, often estimated via Gaussian filtering. The reflection component contains details and color information:

(7) $$\log L(x)=G_{\delta }(x)*\log S(x)$$

where $G_{\delta }(x)$ is a Gaussian kernel with a standard deviation of δ, and ∗ represents the convolution operation.

The reflection component can be expressed as follows:

(8) R(x)=exp[logS(x)−logL(x)]

There is an inherent connection between Retinex theory and atmospheric scattering models; under specific conditions, the two can be related. This paper primarily uses the atmospheric scattering model as the physical basis while drawing on Retinex decomposition concepts for network design.

### 2.2. Convolutional Neural Networks and Transformers

#### 2.2.1. Fundamentals of Convolutional Neural Networks

CNNs perform local sliding window operations through convolutional kernels to extract local features [26]. Key characteristics include local connectivity, weight sharing, and hierarchical structure. For a 2D convolution:

(9) $$(F*K)(i,j)=\sum_{m=-\left\lfloor k/2\right\rfloor }^{\left\lfloor k/2\right\rfloor }\sum_{n=-\left\lfloor k/2\right\rfloor }^{\left\lfloor k/2\right\rfloor }F(i+m,j+n)\cdot K(m,n)$$

where F is the input feature map, K is a convolution kernel of size k×k, and (i,j) is the output position.

Modern CNNs often use batch normalization to accelerate training and improve stability:

(10) $$\hat{x}=\frac{x-\mu }{\sqrt{\sigma^{2}+\varepsilon }},y=\gamma \hat{x}+\beta$$

where μ and σ are the mean and variance of the batch, γ and β are learnable scaling and offset parameters, and ε is the numerical stability constant.

UNet and its variants (UNet++ [27], Attention UNet [28]) are classic architectures in image restoration. Their encoder–decoder structure with skip connections effectively fuses multi-scale information and preserves spatial details:

(11) $$F_{decoder}^{l}=u(F_{decoder}^{l+1})⊕F_{encoder}^{l}$$

where u(⋅) represents upsampling operation, and ⊕ represents feature concatenation or element-wise addition.

#### 2.2.2. Fundamentals of Vision Transformer

Transformer was originally proposed for natural language processing [29]; Vision Transformer (ViT) [30] successfully introduced it to computer vision. The core is the self-attention mechanism, which allows each element to interact with all others, capturing global context.

For an input feature sequence $x\in {\mathbb{R}}^{N\times D}$, self-attention is computed as follows:

(12) $$Attention(Q,K,V)=Soft\max(\frac{QK^{T}}{\sqrt{d_{k}}})V$$

where $Q=XW_{Q}$, $K=XW_{K}$, $V=XW_{V}$ are the query, key, and value matrices obtained by linear transformation of X, $W_{Q},W_{K},W_{V}\in {\mathbb{R}}^{D\times d_{k}}$ are learnable weights, and $d_{k}$ is the dimension of the key vector. The $\sqrt{d_{k}}$ term is used to scale dot products to prevent gradient vanishing.

Multi-head attention executes the above process in parallel multiple times to focus on information representing different subspaces:

(13) $$MultiHead(Q,K,V)=Concat(head_{1},\ldots ,head_{h})W^{O}$$

where $head_{i}=Attention(QW_{i}^{Q},KW_{i}^{K},VW_{i}^{V})$, h is the number of heads.

Transformer blocks typically include multi-head attention layers and feedforward networks (FFN), and employ residual connections and layer normalization:

(14) Z=LayerNorm(X+MultiHead(Q,K,V))

(15) Y=LayerNorm(Z+FFN(Z))

Swin Transformer [31] introduces shifted windows and hierarchical design, reducing computational complexity from $O(N^{2})$ to O(N) while maintaining global modeling capability.

The shift window mechanism achieves cross-window information exchange by alternately using regular window partitioning and shift window partitioning between consecutive Transformer blocks (with an offset of $\left\lfloor M/2\right\rfloor$, where M is the window size):

(16) $$\hat{z}=W-MSA(LN(z^{l-1}))+z^{l-1}$$

(17) $$z^{l}=MLP(LN({\hat{z}}^{l}))+{\hat{z}}^{l}$$

(18) $${\hat{z}}^{l+1}=SW-MSA(LN(z^{l}))+z^{l}$$

where W−MSA represents window multi-head self-attention, and SW−MSA represents shifted window multi-head self-attention.

The Global Transformer Branch (GTB) in this article does not propose a new attention mechanism variant, but is based on the Swin Transformer Tiny architecture [31] for lightweight adaptation, and matches the output of the local CNN branch through feature scale alignment, thereby achieving efficient dual-branch feature fusion.

### 2.3. Object Detection Framework

Object detection aims to locate and classify objects of interest. Single-stage detectors like YOLO series [32], SSD [33], and RetinaNet [34] treat detection as a regression problem, predicting bounding boxes and categories in a single forward pass. YOLOv7 [35] achieves a good balance between speed and accuracy.

Two-stage detectors such as Faster R-CNN [36], Mask R-CNN [37], etc. are used. Firstly, generate Region Proposals, and then classify and perform bounding box regression on each proposed region, which usually has higher accuracy but slower speed. DETR [38] is an end-to-end detector based on Transformer, which abandons manually designed anchor boxes and non-maximum suppression (NMS), but converges slowly.

The loss function of YOLOv7 consists of three parts: bounding box regression loss $L_{box}$, target confidence loss $L_{obj}$, and classification loss $L_{cls}$:

(19) $$L_{YOLOv7}=\lambda_{box}L_{box}+\lambda_{obj}L_{obj}+\lambda_{cls}L_{cls}$$

where bounding box regression usually adopts CIoU Loss [39], which comprehensively considers overlapping areas, center point distance, and aspect ratio:

(20) $$L_{CIoU}=1-IoU+\frac{\rho^{2}(b,b^{gt})}{c^{2}}+\alpha v$$

where ρ is the Euclidean distance, b and $b^{gt}$ are the center points of the predicted box and the true box, c is the diagonal length of the minimum bounding rectangle, v is the term used to measure aspect ratio consistency, and α is the weight coefficient.

Considering the real-time requirements of autonomous driving, we choose the lightweight YOLOv7-tiny as the detection head and integrate it end-to-end with the enhancement backbone.

### 2.4. Attention Mechanism

The core idea of attention mechanisms is to enable models to dynamically focus on important parts of the input [40]. In computer vision, channel attention [41], spatial attention, and their combination (e.g., CBAM [42]) have been widely used for feature recalibration.

Channel attention focuses on “what” is meaningful, and a typical representative is the channel attention module in SENet [41]:

(21) $$z_{c}=\frac{1}{H\times W}\sum_{i=1}^{H}\sum_{j=1}^{W}F_{c}(i,j)$$

(22) $$s=\sigma (W_{2}\delta (W_{1}z))$$

(23) $$F^{\prime }=s⊙F$$

where $z_{c}$ is the global average pooling feature of channel c, $W_{1}$ and $W_{2}$ is the fully connected layer weight, σ is the ReLU activation function, δ is the Sigmoid function, and ⊙ represents channel level multiplication.

Spatial attention focuses on the information-rich region “where”, which can be achieved through spatial attention maps:

(24) $$M_{S}=\sigma (f^{7\times 7}([AvgPool(F);MaxPool(F)]))$$

(25) $$F^{\prime }=M_{S}⊙F$$

where $f^{7\times 7}$ represents a 7×7 convolution operation.

Mixed attention, such as CBAM [42], combines channel and spatial attention, first performing channel attention and then executing spatial attention, achieving more comprehensive feature recalibration.

The innovation of this paper lies not in proposing new attention variants, but in creatively combining cross-attention with physical priors and dual-branch features to design the Physical Fusion Module (PFM).

### 2.5. Chapter Summary

This chapter systematically introduced the core theoretical foundations of PGT-Net, including physical models for low-light image formation, CNN and Transformer principles, object detection frameworks, and attention mechanisms. These theories provide a solid basis for the network design presented in subsequent chapters.

## 3. Overall Architecture of PGT-Net Network

### 3.1. Overall Design Concept

The core design philosophy of PGT-Net is “physical guidance, dual stream complementarity, and end-to-end optimization”. This article aims to build a unified framework that follows physical laws while fully utilizing the powerful representation learning capabilities of deep neural networks.

**Physical guidance:** By introducing a learnable atmospheric scattering physics branch, we inject clear physical prior knowledge into the network, constraining learning within a reasonable physical space and enhancing interpretability and generalization.

**Dual-stream complementarity:** A parallel local CNN branch and Global Transformer Branch work together. The CNN branch excels at capturing local details, while the Transformer branch models long-range dependencies to correct global uneven illumination. The two are fused via a carefully designed mechanism for “local refinement and global coordination”.

**End-to-end optimization:** Seamlessly connecting the enhancement module with the detection module, a unified multi-task loss ensures that enhancement directly serves detection performance.

### 3.2. Overall Network Framework

**Figure 1:**
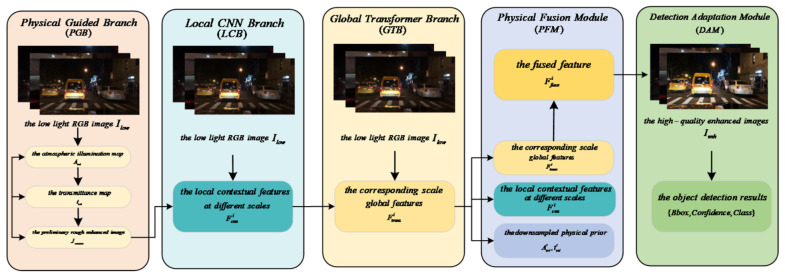
The overall architecture of PGT-Net.

The overall architecture of PGT-Net is shown in Figure 1, consisting of five core components: Physical Guided Branch (PGB), local CNN branch (LCB), Global Transformer Branch (GTB), Physical Fusion Module (PFM), and Detection Adaptation Module (DAM). The entire network works collaboratively in a forward propagation manner.

**Forward propagation process:**

**(1) Input:** A low-light RGB image $I_{low}\in {\mathbb{R}}^{H\times W\times 3}$.

**(2) Physical Guided Branch (PGB):** Input $I_{low}$, estimate the atmospheric illumination map $A_{est}\in {\mathbb{R}}^{H\times W\times 3}$ and transmittance map $t_{est}\in {\mathbb{R}}^{H\times W\times 1}$ of the entire image through a lightweight encoder–decoder network. Meanwhile, based on the atmospheric scattering model, a preliminary rough enhanced image $J_{coarse}$ is calculated as the physical prior.

The original intention of its design is to provide a “navigation device” that conforms to physical laws for the network. Unlike traditional methods that treat atmospheric light A and transmittance t as global constants or heuristic results, we use a learnable lightweight network to estimate the spatial variations in A and t. The advantage of this approach is that it preserves the physical constraints of the atmospheric scattering model on the image formation process (see Section 4.1 for physical consistency loss), while adapting to the spatial variations in lighting and depth in complex traffic scenes through data-driven methods, which is key to improving the model’s generalization ability (see Section 5.5 for generalization testing).

**(3) Dual-branch feature extraction:**

Local CNN branch (LCB): Taking $I_{low}$ as input, the improved UNet++ architecture extracts rich local contextual features $F_{cnni=1}^{i\;N}$ at N different scales, where $F_{cnn}^{i}\in {\mathbb{R}}^{H_{i}\times W_{i}\times C_{i}}$.

Global Transformer Branch (GTB): Divide $I_{low}$ into non-overlapping patches, input them into a Swin Transformer-based encoder, and extract features $F_{transi=1}^{i\;\;N}$ with a global receptive field, whose scale corresponds to the LCB features.

The ideal low-light enhancement requires both local detail repair and global illumination correction. Therefore, this article designs parallel local CNN branches and Global Transformer Branches. The CNN branch, with its inherent inductive bias (local connections, weight sharing), excels at capturing and restoring fine textures of images, such as road sign edges and pedestrian contours; the Transformer branch, through its self-attention mechanism, can effectively model the dependency relationships between distant pixels, thereby correcting global degradation caused by uneven lighting (such as car light halo and large area shadows). This dual-branch design with clear division of labor aims to achieve “each performing its own duties and complementary advantages”, and its effectiveness will be verified through the ablation experiment in Section 5.2 (see Table 3, comparing models A and C).

**(4) Physical Fusion Module (PFM):** This is the core of feature fusion. At each corresponding feature scale i, PFM receives three inputs: $F_{cnn}^{i},F_{trans}^{i}$, and physical priors $A_{est}^{i},t_{est}^{i}$ downsampled from the physical guidance branch to the corresponding scale. PFM achieves deep interaction and fusion of local features, global features, and physical priors through a cross-attention mechanism and adaptive weighting, and outputs the fused feature $F_{fuse}^{i}\in {\mathbb{R}}^{H_{i}\times W_{i}\times C_{i}^{\prime }}$. The decoder part upsamples and aggregates these multi-scale fusion features to ultimately generate high-quality enhanced images $I_{enh}\in {\mathbb{R}}^{H\times W\times 3}$.

Simple feature concatenation or addition cannot fully utilize the guiding role of physical priors. Therefore, we designed a PFM based on cross-attention, which allows physical priors (A, t) to actively “query” and “align” relevant information in local and global features, achieving deep interaction among the three. This design ensures that the final enhancement result not only visually conforms to physical laws, but also preserves rich details in data-driven features. Its superiority is demonstrated through the comparison of models D and E in Section 5.2.

**(5) Detection Adaptation Module (DAM):** takes the enhanced image $I_{enh}$ as input and feeds it into a lightweight YOLOv7 detection head. The detection head utilizes its backbone, neck, and head structures to directly predict the bounding box coordinates, confidence levels, and category probabilities of all targets in the image.

**(6) Output:** The final output of the network is the enhanced image $I_{enh}$ and a set of object detection results {Bbox,Confidence,Class}.

The mathematical representation of the entire network can be summarized as follows:

(26) $$A_{est},t_{est}=F_{PGB}(I_{low})$$

(27) $${\left\{F_{cnn}^{i}\right\}}_{i=1}^{N}=F_{LCB}(I_{low})$$

(28) $${\left\{F_{trans}^{i}\right\}}_{i=1}^{N}=F_{GTB}(I_{low})$$

(29) $$I_{enh}=F_{PFM}(\{F_{cnn}^{i}\},\{F_{trans}^{i}\},\{A_{est}^{i},t_{est}^{i}\})$$

(30) $$\left\{B,C,P\}=F_{DAM}(I_{enh}\right)$$

where $F_{PGB},F_{LCB},F_{GTB},F_{PFM},F_{DAM}$ represents the function mapping of each module, while B,C,P represent bounding boxes, confidence, and class probabilities, respectively.

### 3.3. Physical Guidance Branch (PGB)

The role of the physical guidance branch is to provide a “compass” for the network that conforms to physical laws. It does not directly generate the final output, but rather provides constraints and guidance for subsequent feature fusion. Its design is based on a learnable version of the atmospheric scattering model.

This branch is a small, fully convolutional network, consisting of several downsampling and upsampling layers. It outputs two images with the same resolution as the input:

**Atmospheric illumination map** $A_{est}$: Estimates the ambient illumination intensity at each pixel location. In traffic scenes, values near light sources (car headlights, street lights) are high, while values in shadow areas are low. The network is encouraged to learn the spatial variation in illumination, rather than the constant values in traditional models.

**Transmittance map** $t_{est}$: Estimate the attenuation degree of light propagation in the scene. It is usually related to the scene depth and medium density, with a smaller value of t in distant or heavily fogged areas.

This article also calculates a coarse enhancement result $J_{\mathrm{co}arse}$ based on a physical model:

(31) $$J_{coarse}(x)=\frac{I_{low}(x)-A_{est}(x)}{\max(t_{est}(x),\varepsilon )}+A_{est}(x)$$

where $\varepsilon =10^{-6}$ is a small constant to prevent division by zero. $J_{\mathrm{co}arse}$ usually contains noise and artifacts, but generally reflects the enhancement direction expected by the physical model. $A_{est}$, $t_{est}$ and $J_{\mathrm{co}arse}$ will be passed to the Physical Fusion Modules at different scales as prior information to guide feature fusion.

### 3.4. Dual-Branch Enhanced Backbone

#### 3.4.1. Local CNN Branch (LCB)

The local CNN branch is improved based on the UNet++ architecture, with the main task of capturing and restoring image details. We have made the following key improvements:

**Dense skip connections:** Utilizing the nested dense connection structure of UNet++, it enables the features of each layer in the encoder to directly flow to the corresponding layer and all shallower layers in the decoder. This promotes the reuse and fusion of multi-scale features, facilitating the restoration of finer textures.

**Depthwise separable convolution:** Replacing standard convolution with depthwise separable convolution in some convolutional layers can significantly reduce parameters and computation while maintaining performance.

**Spatial attention module:** Embedding a lightweight spatial attention module on the skip connection path enables the network to adaptively focus on regions with richer information (such as edges and regions with complex textures) and suppress smooth or degraded regions.

This branch outputs a multi-scale local feature pyramid ${\left\{F_{cnn}^{i}\right\}}_{i=1}^{N}$, where N=5 and the corresponding downsampling rates are 1, 2, 4, 8, and 16, respectively.

#### 3.4.2. Introduction to Global Transformer Branch (GTB)

The Global Transformer Branch utilizes Swin Transformer as its backbone, aiming to model global context and address issues such as uneven illumination, extensive shadows, and glare.

Patch partition and embedding: Divide the input image into non-overlapping patches (e.g., 4 × 4 in size) and project them into the feature space through a linear embedding layer.

Hierarchical Swin Transformer blocks: The network comprises multiple stages, with each stage consisting of several Swin Transformer blocks and a patch merging operation. The Swin Transformer block employs self-attention computation based on a shifted window, enabling efficient global information exchange. Patch merging reduces resolution while increasing the number of channels, forming a feature pyramid.

Lightweight design: To balance performance and efficiency, we adopted a “Tiny” version of the configuration, reducing the number of blocks and the dimension of hidden layers, and removing the last stage to align the output feature scale with LCB.

This branch outputs multi-scale global features ${\left\{F_{trans}^{i}\right\}}_{i=1}^{N}$ corresponding to the spatial resolution of LCB.

### 3.5. Physical Fusion Module (PFM) and Decoder

The Physical Fusion Module serves as a bridge connecting physical priors, local features, and global features. For the *i*-th scale:

**Input:** $F_{cnn}^{i}\in {\mathbb{R}}^{H_{i}\times W_{i}\times C_{i}},F_{trans}^{i}\in {\mathbb{R}}^{H_{i}\times W_{i}\times C_{i}},A_{est}^{i}\in {\mathbb{R}}^{H_{i}\times W_{i}\times 3},t_{est}^{i}\in {\mathbb{R}}^{H_{i}\times W_{i}\times 3}$

**Physical prior embedding:** After concatenating $A_{est}^{i}$ and $t_{est}^{i}$, a 1 × 1 convolution is applied to generate physical guidance features $F_{phy}^{i}\in {\mathbb{R}}^{H_{i}\times W_{i}\times C_{i}}$

**Cross-attention fusion: a. Fusion with physics as the query:** Taking $F_{phy}^{i}$ as the query (Q), and concatenating $F_{cnn}^{i}$ and $F_{trans}^{i}$ as the key (K) and value (V) for cross-attention computation. This enables the physical prior to actively retrieve relevant information from local and global features. **b. Inter-feature cross-attention:** Perform bidirectional cross-attention between the output from the previous step and $F_{cnn}^{i}$ and $F_{trans}^{i}$ respectively, to promote complementarity and alignment between local and global features.

**Adaptive weighted fusion:** Finally, through a learnable weight map, the local and global features enhanced by cross-attention are weighted and summed at the pixel level to obtain the fused feature $F_{fuse}^{i}$ for that scale.

The decoder part adopts a progressive upsampling structure. Starting from the deepest layer $F_{fuse}^{N}$, spatial resolution is gradually restored through upsampling, concatenation with $F_{fuse}^{i-1}$ of adjacent scales, and convolution. Finally, a normalized enhanced image $I_{enh}$ is output through a 3 × 3 convolution and Sigmoid activation function.

The decoding process can be represented as follows:

(32) $$F_{dec}^{i}=\{\begin{array}{cc} F_{fuse}^{N}, & i=N\\ H([U(F_{dec}^{i+1}),F_{fuse}^{i}]), & i=N-1,\ldots ,1 \end{array}$$

where U(⋅) denotes bilinear upsampling, H(⋅) denotes convolution operation, and [⋅] denotes feature concatenation.

Final enhanced image generation:

(33) $$I_{enh}=\sigma (W_{out}*F_{dec}^{1}+b_{out})$$

where $W_{out}$ is a 3 × 3 convolution kernel, $b_{out}$ is the bias term, and σ is the Sigmoid activation function.

### 3.6. Detection and Adaptation Module (DAM)

The Detection Adaptation Module directly adopts the detection head structure of YOLOv7-tiny, but its backbone input is replaced with our enhanced image $I_{enh}$. In end-to-end training, the gradient of the detection head can be backpropagated to the entire enhanced backbone and physical guidance branch, thereby guiding the enhancement process to learn more favorable image characteristics for detection.

The detection head of YOLOv7-tiny comprises three main components:

Backbone: Extracts multi-scale features and is typically composed of the CSPNet [43] structure.

Neck: Feature Pyramid Networks (FPNs) [44] and Path Aggregation Networks (PANs) [45,46] are used for multi-scale feature fusion.

Head: Predict bounding boxes, confidence scores, and categories.

The output of the detection head can be expressed as follows:

(34) $$B,C,P=F_{YOLOv7-tiny}(I_{enh})$$

where $B\in {\mathbb{R}}^{K\times 4}$ represents the coordinates of K prediction boxes, $C\in {\mathbb{R}}^{K}$ represents the confidence, and $P\in {\mathbb{R}}^{K\times M}$ represents the probability distribution of M categories.

To further enhance efficiency, we fine-tuned the neck part of YOLOv7-tiny to better adapt to the feature characteristics (e.g., less noise, better contrast) outputted from the PGT-Net enhanced backbone. Simultaneously, we retained YOLOv7’s “trainable bag-of-freebies” strategies, such as label assignment optimization and loss function improvement, to maintain its high performance.

### 3.7. Summary of This Chapter

This chapter elaborated on the overall architecture of PGT-Net. The network integrates physical priors, extracts complementary features via parallel CNN and Transformer branches, and fuses them deeply via PFM, ultimately generating high-quality enhanced images and driving high-performance object detection.

## 4. Design and Implementation of the Network Core Module

### 4.1. Detailed Design of Physical Guided Branch (PGB)

**Figure 2:**
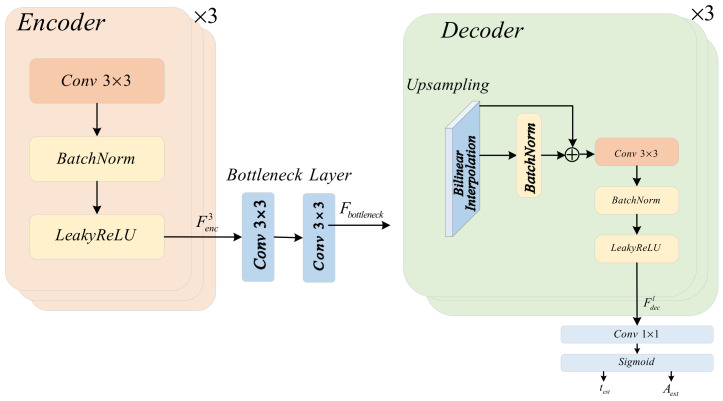
Physical guidance branch (PGB) architecture diagram.

The structure of the physical guidance branch is a lightweight encoder–decoder network, as illustrated in Figure 2. Its objective is to swiftly and stably estimate two pivotal physical parameters that conform to the atmospheric scattering model.

The encoder part consists of three downsampling blocks. Each block includes: a 3 × 3 standard convolutional layer (with a stride of 2 for downsampling), a BatchNorm layer, and a LeakyReLU activation function (with a negative slope of 0.2). The encoder gradually downsamples the input image to 1/8 of its original size, extracting high-level semantics while compressing spatial information, which is used to estimate global illumination distribution and depth-related transmittance.

The feature computation of the l-th layer encoder is as follows:

(35) Fencl=LeakyReLU(BN(Conv3×3,stride=2(Fencl−1)))

where $F_{enc}^{0}=I_{low}$.

The bottleneck layer consists of two consecutive 3 × 3 convolutional layers, which are used to further process the encoded features:

(36) Fbottleneck=LeakyReLU(BN(Conv3×3(Fenc3)))

The decoder part corresponds to each scale of the encoder and consists of three upsampling blocks. Each block first upsamples the feature map by a factor of two using bilinear interpolation, and then concatenates the features of the corresponding scale from the encoder through skip connections. The concatenated features pass through a 3 × 3 convolution, BatchNorm, and LeakyReLU.

The feature computation for the l-th layer decoder is as follows:

(37) Fdecl=LeakyReLU(BN(Conv3×3([U(Fdecl+1),Fenc3−l])))

where U(⋅) denotes bilinear upsampling, and [⋅] denotes feature concatenation.

The last two layers of the decoder are used to predict the following two physical quantities, respectively:

A 1 × 1 convolution + Sigmoid, outputting a single-channel transmittance map $t_{est}$ (value range [0, 1]).

A 1 × 1 convolution + Sigmoid, outputting a three-channel atmospheric illumination map $A_{est}$ (value range [0, 1]).

Mathematically expressed as follows:

(38) $$t_{est}=\sigma (W_{t}*F_{dec}^{l}+b_{t})$$

(39) $$A_{est}=\sigma (W_{A}*F_{dec}^{l}+b_{A})$$

where $W_{t}\in {\mathbb{R}}^{1\times 1\times C\times 1}$, $W_{A}\in {\mathbb{R}}^{1\times 1\times C\times 3}$ are convolution kernels, $b_{t}$, $b_{A}$ are bias terms, and σ is the Sigmoid function.

In this paper, $A_{est}$ is initialized as a constant map close to the global mean of the input image to accelerate convergence:

(40) $$A_{est}^{init}=\frac{1}{HW}\sum_{x}I_{low}(x)\cdot 1_{H\times W}$$

Physical consistency loss: To ensure that the output of PGB conforms to the physical model, a physical reconstruction loss $L_{phy}$ is introduced:

(41) $$L_{phy}=\frac{1}{HW}\sum_{x=1}^{H}\sum_{y=1}^{W}||I_{low}(x,y)-[J_{coarse}(x,y)\cdot t_{est}(x,y)+A_{est}(x,y)\cdot (1-t_{est}(x,y))]|{|}_{1}$$

where $||\cdot |{|}_{1}$ denotes the L1 norm. This loss mandates that the relationship between $A_{est}$, $t_{est}$, and $J_{coarse}$ adheres to the atmospheric scattering model (Equation (1)), thereby providing strong physical constraints for the learning of the branches.

### 4.2. Improved Local CNN Branch (LCB)

**Figure 3:**
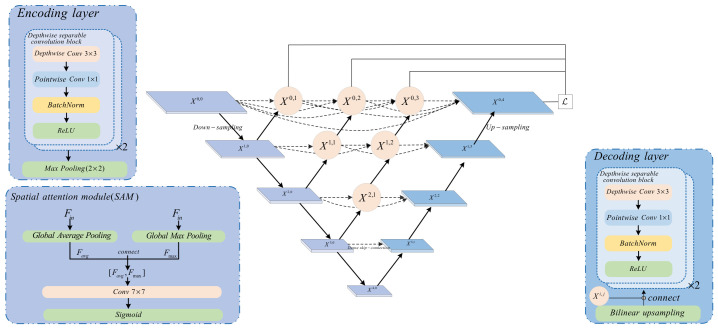
Improved local CNN branch (LCB) architecture diagram.

Based on UNet++, the local CNN branch incorporates depthwise separable convolution and a spatial attention mechanism, with the specific structure illustrated in Figure 3.

Main architecture: A five-layer encoder–decoder structure is adopted. Each layer of the encoder comprises two depthwise separable convolution blocks (each block: Depthwise Conv 3 × 3 → Pointwise Conv 1 × 1 → BN → ReLU), with channel numbers of 32, 64, 128, 256, and 512, respectively. Then, 2 × 2 max pooling is performed for downsampling after each layer.

The computation of depthwise separable convolution can be decomposed into depthwise convolution and pointwise convolution:

(42) $$F_{dw}=K_{dw}⊗F_{in}$$

(43) $$F_{out}=K_{pw}\times F_{dw}$$

where ⊗ denotes depthwise convolution (with each input channel being convolved separately), and × denotes standard convolution.

In each layer of the decoder, the resolution is first doubled through bilinear upsampling, and then concatenated with feature maps from different encoding layers (via dense skip connections). Finally, feature fusion is performed through two depthwise separable convolution blocks.

Dense skip connection: This is the core of UNet++. Assuming $X_{i,j}$ represents the node feature between the i-th encoder layer and the j th decoder layer (where i is the index of the encoding layer and j is the index of the decoding layer), the feature calculation is as follows:

(44) $$X^{i,j}=\{\begin{array}{cc} H(X^{i-1,j}), & if\;j=0\\ H([X^{i,j-1},U(X^{i+1,j-1})]), & if\;j>0 \end{array}$$

where H(⋅) denotes the convolution operation, U(⋅) denotes upsampling, and [⋅] denotes concatenation. This structure forms a dense feature pyramid, enhancing gradient flow and information reuse.

Spatial attention module (SAM): Embedded on the critical skip connection path. This module first performs global average pooling and global max pooling on the input feature $F_{in}\in {\mathbb{R}}^{H\times W\times C}$ to obtain two spatial descriptors. After concatenating them, a 7 × 7 convolution and Sigmoid activation function are applied to generate a spatial attention weight map $W_{spatial}\in {\mathbb{R}}^{H\times W\times 1}$.

It is mathematically expressed as follows:

(45) $$F_{avg}=\frac{1}{C}\sum_{c=1}^{C}F_{in}(:,:,c)$$

(46) $$F_{\max}=\underset{c}{\max}\;F_{in}(:,:,c)$$

(47) $$W_{spatial}=\sigma (Conv_{7\times 7}([F_{avg},F_{\max}]))$$

The final output is $F_{out}=F_{in}⊗W_{spatial}$, where ⊗ denotes element-wise multiplication. This enables the network to adaptively emphasize the features of important regions.

### 4.3. Global Transformer Branch (GTB)

The Global Transformer Branch is tailored based on the Swin Transformer Tiny architecture, with its configuration shown in Table 2.

GTB adopts Swin Transformer Tiny configuration, with 2, 2, and 6 Transformer blocks for each stage, 96, 192, and 384 channels, 3, 6, and 12 attention heads, and a window size of 7×7. Patch Embedding uses 4×4 convolution with an output dimension of 96:

(48) $$z_{0}=[x_{p}^{1}E;x_{p}^{2}E;\ldots ;x_{p}^{N}E]$$

where $x_{p}^{i}\in {\mathbb{R}}^{48}$ represents the i-th patch, $E\in {\mathbb{R}}^{48\times 96}$ denotes the projection matrix, and $E\in {\mathbb{R}}^{48\times 96}$ signifies the number of patches.

Swin Transformer block: Each block comprises a window-based multi-head self-attention (W-MSA or SW-MSA) module and a two-layer MLP, with LayerNorm and residual connections in between.

For windowed multi-head self-attention, the input features are first divided into non-overlapping M×M windows (M = 7). For each feature $z\in {\mathbb{R}}^{M^{2}\times d}$ within each window, self-attention is calculated:

(49) $$Attention(Q,K,V)=Soft\max(\frac{QK^{T}}{\sqrt{d}}+B)V$$

where $B\in {\mathbb{R}}^{M^{2}\times M^{2}}$ is a learnable relative position bias.

The sliding window mechanism achieves cross-window information interaction by alternating between regular window partitioning and sliding window partitioning (with an offset of $\left\lfloor M/2\right\rfloor$) between consecutive blocks.

Patch merging: Performed at the beginning of Stage 2 and 3, it concatenates adjacent 2 × 2 patch features, multiplies the number of channels by 2 through a linear layer, and simultaneously reduces the resolution by a factor of 2:

(50) $$z^{\prime }=Linear(Concat(z_{0,0},z_{0,1},z_{1,0},z_{1,1}))$$

where $z_{i,j}$ represents the features in a 2 × 2 neighborhood.

Output features: We take the feature maps at the end of Stage 1, 2, and 3 as the multi-scale global feature outputs $F_{trans}^{1},F_{trans}^{2},F_{trans}^{3}$, with resolutions being 1/4, 1/8, and 1/16 of the input, respectively. To align with the 5-layer features of LCB, we additionally extract one feature before Stage 1 (after PatchEmbed) and one after Stage 3 (downsampled by a factor of 2 through a 3 × 3 convolution), forming a feature pyramid with 5 scales.

### 4.4. Detailed Implementation of the Physical Fusion Module (PFM)

PFM is the key to feature fusion, for the i-th scale:

**1. Input pre-processing**: $F_{cnn}^{i},F_{trans}^{i}$ are first aligned in channels through a 1 × 1 convolution (aligned to D = 128 dimensions):

(51) $${\hat{F}}_{cnn}^{i}=W_{cnn}^{i}*F_{cnn}^{i}+b_{cnn}^{i}$$

(52) $${\hat{F}}_{trans}^{i}=W_{trans}^{i}*F_{trans}^{i}+b_{trans}^{i}$$

The physical priors $A_{est}^{i}$ and $t_{est}^{i}$ are concatenated and passed through a 1 × 1 convolution to generate physical guidance features $F_{phy}^{i}\in {\mathbb{R}}^{H_{i}\times W_{i}\times D}$:

(53) $$F_{phy}^{i}=W_{phy}^{i}*[A_{est}^{i},t_{est}^{i}]+b_{phy}^{i}$$

**2. Physics-Guided Cross-Attention (PGCA):**

Take $F_{phy}^{i}$ as the query (Q), and use the concatenation of ${\hat{F}}_{cnn}^{i}$ and ${\hat{F}}_{trans}^{i}$ as the key (K) and value (V).

Calculate cross-attention:

(54) $$Attn_{pg}^{i}=Soft\max(\frac{Q{\left(K\right)}^{T}}{\sqrt{d_{k}}})V$$

where $Q=reshape(F_{phy}^{i})\in {\mathbb{R}}^{N_{i}\times D}$, $K=reshape({\hat{F}}_{cnn}^{i},{\hat{F}}_{trans}^{i})\in {\mathbb{R}}^{2N_{i}\times D}$, V=K.

**3. Local-Global Cross-Attention (LGCA):**

Use $Attn_{pg}^{i}$ as the query, and perform bidirectional cross-attention with ${\hat{F}}_{cnn}^{i}$ and ${\hat{F}}_{trans}^{i}$ respectively:

(55) $$Attn_{cnn}^{i}=Soft\max(\frac{Attn_{pg}^{i}{\left({\hat{F}}_{cnn}^{i}\right)}^{T}}{\sqrt{d_{k}}}){\hat{F}}_{cnn}^{i}$$

(56) $$Attn_{trans}^{i}=Soft\max(\frac{Attn_{pg}^{i}{\left({\hat{F}}_{trans}^{i}\right)}^{T}}{\sqrt{d_{k}}}){\hat{F}}_{trans}^{i}$$

**4. Adaptive weighted fusion:**

By concatenating $Attn_{cnn}^{i}$ and $Attn_{trans}^{i}$, a two-channel weight map $W^{i}\in {\mathbb{R}}^{H_{i}\times W_{i}\times 2}$ is generated through a small convolutional network:

(57) Wi=Soft maxdim=2(Conv3×3(ReLU(Conv3×3([Attncnni,Attntransi]))))

where $Soft\;\max_{\dim=2}$ denotes performing Softmax operation in the channel dimension.

Final fused features:

(58) $$F_{fuse}^{i}=W_{:,:,0}^{i}⊙Attn_{cnn}^{i}+W_{:,:,1}^{i}⊙Attn_{trans}^{i}$$

where ⊙ denotes element-wise multiplication.

### 4.5. Multi-Task Loss Function

The end-to-end training of PGT-Net is jointly supervised by four loss terms:

Physical consistency loss ($L_{phy}$): As defined in Formula (41), it constrains the physical guidance branch.

Image quality loss ($L_{quality}$): ensures pixel-level and perceptual fidelity of the enhanced image.

$L_{1}$ **reconstruction loss:** measures pixel-level error and is more robust to outliers:

(59) $$L_{rec}=\frac{1}{3HW}\sum_{c=1}^{3}\sum_{x=1}^{H}\sum_{y=1}^{W}|I_{enh}^{\left(c\right)}(x,y)-I_{gt}^{\left(c\right)}(x,y)|$$

**Perception loss:** Feature similarity based on pre-trained VGG-19 network:

(60) $$L_{per}=\frac{1}{C_{j}H_{j}W_{j}}{\left\|\phi_{j}(I_{enh})-\phi_{j}(I_{gt})\right\|}_{2}^{2}$$

where $\phi_{j}(\cdot )$ is the feature extractor of the j-th layer of the VGG-19 network, and $C_{j}\times H_{j}\times W_{j}$ are the dimensions of the features in this layer.

**SSIM loss:** Structural Similarity Index loss

(61) $$L_{ssim}=1-\frac{1}{M}\sum_{m=1}^{M}SSIM(P_{m}(I_{enh}),P_{m}(I_{gt}))$$

where $P_{m}(\cdot )$ represents the m-th local image block, and M is the number of blocks. The SSIM is calculated as follows:

(62) $$SSIM(x,y)=\frac{\left(2\mu_{x}\mu_{y}+C_{1})(2\sigma_{xy}+C_{2}\right)}{\left(\mu_{x}^{2}+\mu_{y}^{2}+C_{1})(\sigma_{x}^{2}+\sigma_{y}^{2}+C_{2}\right)}$$

where $\mu_{x}$, $\mu_{y}$ are the means, $\sigma_{x}^{2}$, $\sigma_{y}^{2}$ are the variances, $\sigma_{xy}$ is the covariance, and $C_{1}$, $C_{2}$ are stability constants.

**Total image quality loss:**

(63) $$L_{quality}=\lambda_{rec}L_{rec}+\lambda_{per}L_{per}+\lambda_{ssim}L_{ssim}$$

Detection loss ($L_{\det}$): Utilize the loss function of YOLOv7:

(64) $$L_{\det}=\lambda_{box}L_{box}+\lambda_{obj}L_{obj}+\lambda_{cls}L_{cls}$$

where $L_{box}$ is the CIoU loss [39]:

(65) $$L_{CIoU}=1-IoU+\frac{\rho^{2}(b,b^{gt})}{c^{2}}+\alpha v$$

where ρ represents the Euclidean distance, c denotes the diagonal length of the minimum enclosing rectangle, and v measures the consistency of aspect ratio:

(66) $$v=\frac{4}{\pi^{2}}{\left(\arctan\frac{w^{gt}}{h^{gt}}-\arctan\frac{w}{h}\right)}^{2}$$

(67) $$\alpha =\frac{v}{(1-IoU)+v}$$

$L_{obj}$ represents the binary cross-entropy loss, while $L_{cls}$ denotes the multi-class cross-entropy loss.

Total loss:

(68) $$L_{total}=L_{phy}+L_{quality}+L_{\det}$$

In the experiment, the loss weights were determined through grid search as follows: $\lambda_{rec}=1.0$, $\lambda_{per}=0.1$, $\lambda_{ssim}=0.5$; $\lambda_{box}=0.05$, $\lambda_{obj}=0.7$, $\lambda_{cls}=0.3$.

### 4.6. Training Strategy and Implementation Details

Optimizer: The AdamW optimizer [47] is adopted, with an initial learning rate of $1\times 10^{-4}$ and a weight decay of $1\times 10^{-4}$. The cosine annealing learning rate scheduler is used:

(69) $$\eta_{t}=\eta_{\min}+\frac{1}{2}(\eta_{\max}-\eta_{\min})(1+\cos(\frac{T_{cur}}{T_{\max}}\pi ))$$

where $\eta_{\max}=1\times 10^{-4}$, $\eta_{\min}=1\times 10^{-6}$, $T_{cur}$ represents the current iteration count, and $T_{\max}$ denotes the total iteration count. A total of 300 epochs are trained.

**Key convolutional parameters:** In the local CNN branch, depthwise separable convolutions use 3 × 3 depthwise kernels and 1 × 1 pointwise kernels. In the physical guidance branch, all standard convolutions use 3 × 3 kernels with stride 2 for downsampling. The spatial attention module uses a 7 × 7 convolution kernel to generate attention weights. In the Physical Fusion Module, cross-attention uses a projection dimension D = 128, and the adaptive weighting network consists of two 3 × 3 convolutional layers (Section 4.4).

Data augmentation: Apply random horizontal flipping (probability 0.5), random rotation (−15° to 15°), and color jittering (adjustment of brightness, contrast, and saturation by ±10% respectively) to the input low-illumination images to enhance model robustness. For training with paired data, apply the same geometric transformations to the clear images.

Gradient clipping: To avoid gradient explosion, the gradient clipping strategy is adopted:

(70) $$if\left\|g\right\|>\theta :g\leftarrow \frac{\theta }{\left\|g\right\|}g$$

where g is the gradient and θ=1.0 is the clipping threshold.

Implementation platform: Utilizing the PyTorch 1.12 framework, distributed data parallel training was conducted on four NVIDIA RTX 3090 GPUs, with a batch size of 16 (four images per GPU). Mixed Accuracy Training (AMP) was employed to accelerate the training process and reduce memory footprint. The training duration was approximately 48 h.

### 4.7. Summary of This Chapter

This chapter detailed the technical implementation of each core module in PGT-Net, including the learnable physical guidance branch, improved UNet++ LCB, lightweight Swin Transformer GTB, and the PFM for deep feature fusion. The multi-task loss and training strategies were also described.

## 5. Experiment and Result Analysis

### 5.1. Experimental Setup

#### 5.1.1. Dataset

This article utilizes three public datasets for training and evaluation:

ExDark [48]: 7363 low-light images, 10 object categories. 70% training, 30% testing.

BDD100K-night [46]: ~20,000 nighttime images from BDD100K. 8:1:1 train/val/test split.

SID (See-in-the-Dark) [49]: RAW dataset for extreme low-light imaging; sRGB subset used for additional quality assessment.

For paired data training, we use the paired subset from ExDark and construct paired data from BDD100K-day using Retinex-based synthesis:

(71) $$I_{syn}=I_{normal}\cdot L_{low}$$

where $L_{low}$ is a randomly generated low-light map with a value range of [0.05, 0.3].

To further validate generalization, we also conduct experiments on the full BDD100K dataset (100K images, 70K/10K/20K split).

During training, we apply standard data augmentation techniques to improve model robustness, including random horizontal flipping (probability 0.5), random rotation (within ±15°), and color jittering (brightness, contrast, and saturation adjustments of ±10%). For paired training data, the same geometric transformations are applied to both low-light and ground-truth clear images to maintain spatial correspondence.

#### 5.1.2. Evaluation Metrics

**1. Image enhancement quality:**

PSNR (Peak Signal-to-Noise Ratio):

(72) $$PSNR=10\cdot \log_{10}(\frac{MAX^{2}}{MSE})$$

where MAX is the maximum pixel value (usually 255) and MSE is the mean squared error:

(73) $$MSE=\frac{1}{3HW}\sum_{c=1}^{3}\sum_{x=1}^{H}\sum_{y=1}^{W}(I_{enh}^{\left(x\right)}(x,y)-I_{gt}^{\left(c\right)}(x,y){)}^{2}$$

The unit is dB, and the higher the value, the better.

SSIM (Structural Similarity Index): As defined in Formula (62), its range is [0, 1], with a higher value indicating better performance.

LPIPS (Learning Perceptual Image Patch Similarity) [50]: Perceptual similarity based on deep features, using pre-trained AlexNet to extract features:

(74) $$LPIPS=\frac{1}{L}\sum_{l=1}^{L}\frac{1}{H_{l}W_{l}}{\sum_{h,w}\left\|w_{l}⊙({\hat{y}}_{hw}^{l}-{\hat{y}}_{0hw}^{l}\right\|}_{2}^{2}$$

where ${\hat{y}}^{l}$ and ${\hat{y}}_{0}^{l}$ represent the features of the l-th layer, and $w_{l}$ denotes the learnable weight, with a lower value indicating a better performance.

**2. Target detection performance:**

mAP@0.5: mean average precision at an Intersection over Union (IoU) threshold of 0.5:

(75) $$AP=\int_{0}^{1}p(r)dr$$

where p(r) represents the precision–recall curve. mAP is the mean average precision (AP) of all categories.

mAP@0.5:0.95: The average mAP with IoU thresholds ranging from 0.5 to 0.95, with a step size of 0.05, indicating a stricter criterion.

FPS (Frames Per Second): The inference speed of processing 512 × 512 images on a single NVIDIA RTX 3090 GPU.

**3. Model complexity:**

Number of parameters (Params): The total number of parameters in the model.

Computation amount (FLOPs): The number of floating-point operations required to process a 512 × 512 image.

Memory usage: The VRAM usage during inference.

#### 5.1.3. Comparison Method

This study selected five representative advanced methods for comparison:

**Traditional:** LIME [10], MF [11] (2025).

**Supervised deep learning:** RetinexNet [6], KinD [7].

**Unsupervised:** Zero-DCE [14], EnlightenGAN [13].

**Transformer-based:** Uformer [15].

**Two-stage:** KinD (enhancement) + YOLOv7 (detection).

**Existing joint method:** JLOD [18].

**Recent lightweight detection-oriented:** YOLOv21 [51] (2024), included in Table 6 to evaluate compatibility with the latest lightweight detection heads.

For methods with complete open source code and pre-trained models (RetinexNet, KinD, Zero-DCE, EnlightenGAN, Uformer), this article prioritizes using their official pre-trained models and fine-tunes them on the target dataset (50 rounds of fine-tuning with a learning rate of $1\times 10^{-5}$).

For methods that do not provide pre-trained models or have architecture mismatches (JLOD), this article retrains them on the same dataset using the code provided by the author and default hyperparameters.

All comparison methods use the same input size (512 × 512), training/testing set partitioning, and evaluation metric calculation scripts as PGT-Net to ensure fair comparison.

#### 5.1.4. Experimental Environment

Hardware: Intel Xeon Gold 6248R CPU, 256 GB RAM, 4× NVIDIA RTX 3090 GPUs (24 GB VRAM).

Hardware: Intel Xeon Gold 6248R, 256 GB RAM, 4× NVIDIA RTX 3090.

Software: Ubuntu 20.04, Python 3.8, PyTorch 1.12.1, CUDA 11.3.

Training: Input size 512 × 512, batch size 16, AdamW ($\beta_{1}$ = 0.9, $\beta_{2}$ = 0.999), initial learning rate is $2\times 10^{-4}$, cosine annealing, warm-up 10 epochs.

### 5.2. Ablation Experiment

This article conducted ablation experiments on the ExDark test set to verify the effectiveness of each module (Table 3).

**Figure 4:**
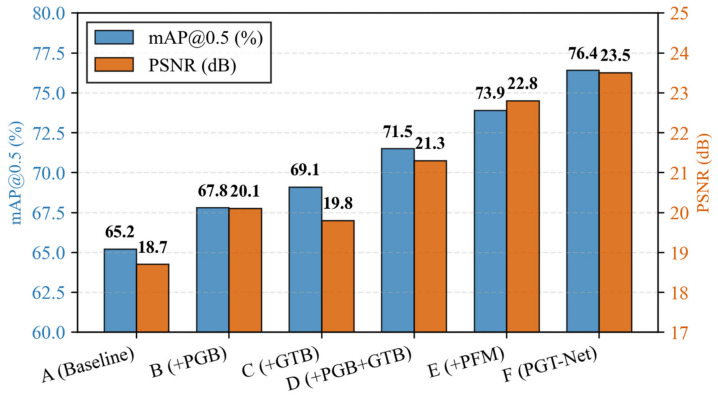
Comparison of key indicators in ablation experiments.

Figure 4 shows the comparison results of key indicators in the ablation experiment, the results of the ablation experiment indicate that:

A vs. B: By solely incorporating the Physical Guided Branch (PGB), both mAP and PSNR exhibit stable improvements (+2.6% mAP, +1.4 dB PSNR), thereby validating the effectiveness of physical priors. Physical priors offer explicit guidance to the network, circumventing the blindness inherent in purely data-driven approaches.

A vs. C: By incorporating only the Global Transformer Branch (GTB) and combining it with LCB, the performance improvement surpasses that of merely adding PGB (+3.9% mAP), indicating the crucial role of global context information in low-light scenarios. The long-range dependency modeling capability of Transformer aids in addressing global illumination unevenness.

B/C vs. D: The simultaneous incorporation of PGB and GTB yielded a notable synergistic effect (1 + 1 > 2), with an enhancement (+6.3% mAP) exceeding the combined individual contributions of the two (2.6 + 3.9 = 6.5), indicating complementarity between physical priors and global features.

D vs. E: After incorporating the Physical Fusion Module (PFM), performance has seen another significant improvement (+2.4% mAP, +1.5 dB PSNR), demonstrating that our designed cross-attention fusion mechanism is far superior to simple feature concatenation or addition. PFM achieves deep interaction between physical priors, local features, and global features.

E vs. F (Full): The introduction of end-to-end training (where the detection loss is directly backpropagated) led to the most significant improvement in mAP (+2.5%), which fully demonstrates that joint optimization makes the enhancement process more task-oriented towards detection. The small improvement in PSNR (+0.7 dB) indicates that end-to-end training greatly optimizes detection-related features while sacrificing some pixel fidelity.

G vs. F (Full): The physical consistency loss not only improves the enhancement quality (PSNR + 0.6 dB), but also significantly improves the detection accuracy (mAP + 1.6%), with reduced standard deviation (from 0.4 to 0.3 for mAP), indicating that this loss helps guide the network to learn more stable and discriminative intermediate features. Qualitatively, incorporating L_phy leads to better suppression of artifacts (e.g., halos around headlights) and more natural texture preservation, further enhancing the positive guidance effect of physical priors on detection tasks.

### 5.3. Comparison of Image Enhancement Quality

**Figure 5:**
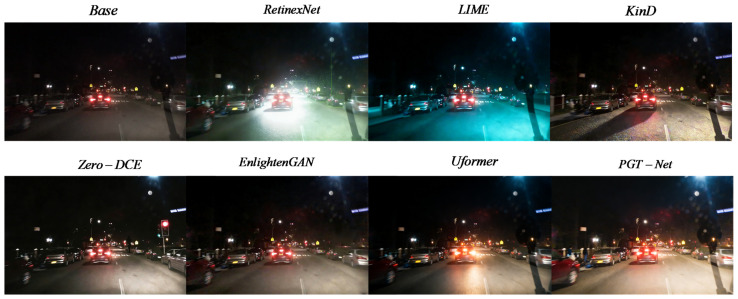
The visual comparison during complex nighttime traffic scenes.

On the ExDark and SID datasets, this article quantitatively compared PGT-Net with mainstream enhancement methods, and the results are presented in Table 4. Figure 5 illustrates the visual comparison during complex nighttime traffic scenes.

PGT-Net achieves the best PSNR and SSIM on both datasets. On LPIPS, it is slightly inferior to Uformer but significantly better than other methods, indicating that the pure Transformer model has a slight advantage in perceptual similarity, while PGT-Net achieves better overall balance through physical priors and local CNNs.

Traditional methods (LIME) show overexposure and noise; RetinexNet has color shifts and halos; Zero-DCE lacks contrast; EnlightenGAN introduces unnatural textures; KinD amplifies noise; Uformer performs well but lacks local highlight suppression. PGT-Net effectively suppresses overexposure, restores distant details, and maintains natural colors and global consistency.

As visually compared in Figure 5, LIME suffers from severe overexposure in strong light source areas and amplifies dark noise; RetinexNet produces noticeable color shifts and halation artifacts; Zero-DCE lacks overall contrast and has limited restoration of dark details; EnlightenGAN generates unnatural texture artifacts and inconsistent brightness; KinD significantly amplifies noise in extremely dark areas; and Uformer performs well in detail restoration but lacks sufficient suppression of local highlights. In contrast, PGT-Net, through a physically guided fusion mechanism, effectively suppresses vehicle light overexposure, restores distant dark details (such as vehicle contours and pedestrians), and maintains natural colors and global consistency, demonstrating comprehensive advantages in both visual quality and quantitative metrics.

Computational efficiency comparison: Table 5 presents the computational complexity of each method.

PGT-Net achieves 31.3 FPS on RTX 3090, meeting real-time requirements. On Jetson AGX Orin (FP16), inference time is 68 ms (14.7 FPS); with TensorRT acceleration, it reaches 42 ms (23.8 FPS), suitable for edge deployment.

### 5.4. Comparison of Object Detection Performance

This article evaluated the performance of enhanced object detection on the more challenging BDD100K-night test set, and the results are presented in Table 6.

**Figure 6:**
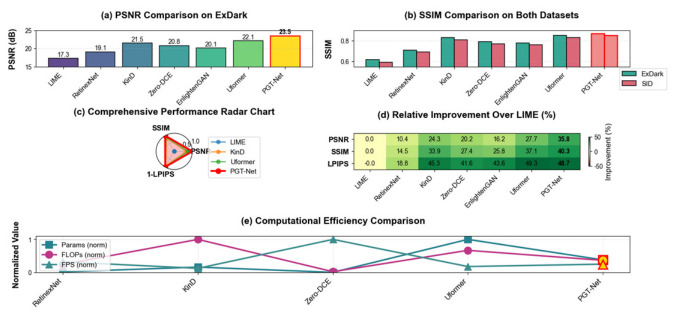
Image enhancement quality comparison. (**a**) PSNR comparison on ExDark dataset, where PGT-Net achieves the highest value (23.5 dB). (**b**) SSIM comparison on both ExDark and SID datasets, showing consistent superiority of PGT-Net. (**c**) Radar chart of normalized PSNR, SSIM, and (1-LPIPS) for four representative methods, demonstrating that PGT-Net achieves the best overall balance. (**d**) Heatmap of relative improvement over the baseline method LIME (in percentage), where PGT-Net obtains substantial gains across all three metrics. (**e**) Normalized computational efficiency (parameters, FLOPs, and FPS) of different models; PGT-Net attains a favorable trade-off between performance and complexity. These results collectively validate the effectiveness of the proposed physically guided Transformer-CNN hybrid architecture.

Figure 6 shows the comparative analysis results of image enhancement quality, any enhancement method can improve detection performance under low illumination, confirming the necessity of pre-enhancement. The baseline mAP@0.5 without enhancement is only 58.7%, indicating that low illumination severely affects detection performance.

To evaluate the adaptability of different lightweight detection heads, this article replaced the detection heads of PGT-Net with YOLOv5-s [52], YOLOv7-tiny [38] and YOLOv8 nano [53], and compared them on the BDD100K night test set. As shown in Table 6, YOLOv7-tiny achieved the highest detection accuracy; mAP@0.5 reached 72.4%, which is 0.8% and 0.5% higher than YOLOv5-s and YOLOv8-nano, respectively. Although YOLOv8 nano has a slightly faster inference speed (115 FPS), its accuracy is relatively low; YOLOv5-s has a fast speed but a significant difference in accuracy. Analyzing the reasons, the “trainable bag-of-freebies” strategy proposed by YOLOv7 [35] has better compatibility with the multi-task loss function proposed in this paper, and can more effectively utilize the features provided by the enhancement module. Therefore, this article ultimately chooses YOLOv7-tiny as the detection head of PGT-Net to achieve the maximum benefit of enhanced detection joint optimization.

**Figure 7:**
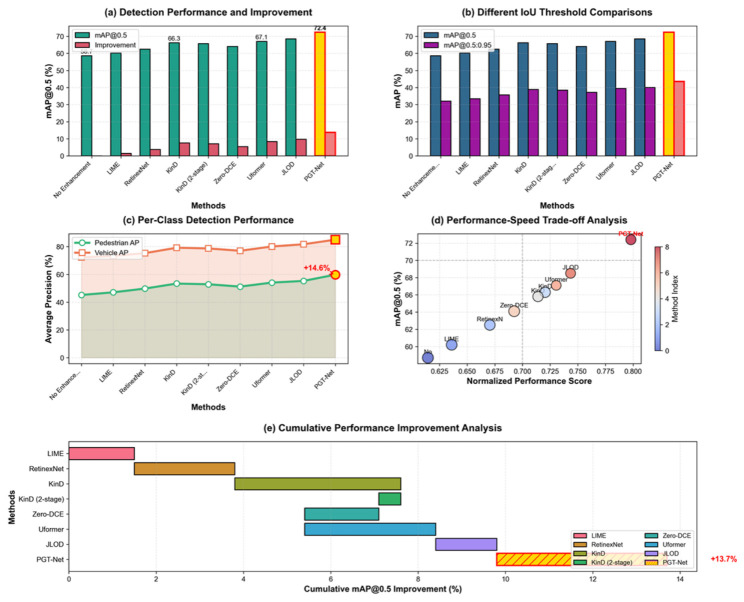
Object detection performance comparison on BDD100K-night. (**a**) Bar chart of mAP@0.5 (%) and absolute improvement over “No Enhancement”. (**b**) Comparison of mAP@0.5 and mAP@0.5:0.95. (**c**) Per-class Average Precision for pedestrian and vehicle detection. (**d**) Scatter plot of normalized performance score vs. mAP@0.5; bubble size indicates FPS, showing the performance-speed trade-off. (**e**) Cumulative mAP@0.5 improvement from “No Enhancement” to each method, with PGT-Net highlighted in gold. PGT-Net consistently outperforms all other methods across all metrics, achieving the highest detection accuracy while maintaining competitive real-time speed (102 FPS).

To further evaluate the adaptability of PGT-Net to different lightweight detection heads, we replaced the detection head with YOLOv5-s [53], YOLOv7-tiny [35], YOLOv8-nano [54], and the recently proposed lightweight-oriented YOLOv21 [51]. As shown in Table 6, the combination of PGT-Net with YOLOv7-tiny achieves the highest detection accuracy (72.4% mAP@0.5), while the combination with YOLOv21 achieves the highest inference speed (128 FPS), highlighting its potential for latency-critical applications. The YOLOv21 variant, designed with a more efficient architecture for edge devices, demonstrates a favorable speed–accuracy trade-off. However, given that the primary goal of this work is to maximize detection accuracy while maintaining real-time performance (≥30 FPS), we select PGT-Net + YOLOv7-tiny as our default model for its superior accuracy. This choice also facilitates a fair comparison with the state-of-the-art methods, many of which also utilize YOLOv7 as their detection head. Figure 7 shows a comparison of object detection performance on the BDD100K night dataset, the results confirm that PGT-Net’s enhancement module is compatible with a range of lightweight detectors and consistently provides significant performance gains.

A detailed category analysis reveals that PGT-Net achieves a particularly significant improvement in pedestrian detection (59.8% AP), which is 14.6% higher than that without enhancement. Pedestrians are typically more difficult to detect under low-illumination conditions, and PGT-Net’s physical guidance and global correction capabilities are particularly effective in this regard.

Compared to Uformer, which also utilizes Transformer, PGT-Net leads in detection accuracy by 5.3% and boasts a higher FPS (102 vs. 95), thanks to our efficient hybrid architecture design.

Compared to the two-stage approach (KinD independent training followed by detector), our end-to-end approach (PGT-Net) achieves superior performance (72.4% vs. 65.8%) with comparable FPS (102 vs. 105), demonstrating the efficiency of joint optimization. End-to-end training enables the enhancement module to learn detection-friendly features.

The FPS of PGT-Net is 102, satisfying the real-time processing requirement (>30 FPS) and achieving an excellent balance between accuracy and speed.

### 5.5. Analysis of the Impact Mechanism of Enhanced Modules

To distinguish the sources of improved detection performance, this article designed the following comparative experiments:

Only image quality path: Use the PGT-Net enhanced image as input, but freeze the enhancement module parameters and only train the detection head (i.e., two-stage mode). At this moment, mAP@0.5 70.1%, lower than end-to-end training (72.4%).

Feature-only representation path: Directly input the feature map output by the enhancement module (rather than the enhanced image) into the detection head, bypassing the image domain reconstruction. At this moment, mAP@0.5 71.6%, higher than the two-stage mode but still lower than end-to-end.

End-to-end complete mode: mAP@0.5 reaches 72.4%.

The above results indicate that the improvement in image quality (two-stage) contributes approximately +11.4% mAP gain (compared to 58.7% without enhancement), while the additional feature alignment brought by end-to-end joint optimization contributes +2.3% gain. To further verify, this article calculated the mutual information between the output features of the enhancement module and the deep features of the detection head. In the end-to-end training mode, the mutual information value (0.73) was significantly higher than that in the two-stage mode (0.58), indicating that joint training enabled the enhancement module to learn feature representations that are more conducive to the detection task. Therefore, the improvement in detection performance not only comes from clearer images, but also from task-oriented feature learning.

### 5.6. Generalization Ability Test

To verify the generalization ability of PGT-Net in unknown scenarios and extreme conditions, this article conducted cross-dataset testing:

**Cross-sensor**: Model trained on ExDark tested on FLIR thermal imaging dataset; PGT-Net shows more stable performance than KinD and Uformer (mAP drop −8.2% vs. −15.3%).

**Extreme conditions**: Tunnel exit glare and rainy night reflection scenes; PGT-Net handles exposure balance and raindrop suppression better.

**Domain adaptation**: Style transfer [55] to other city datasets; PGT-Net shows superior generalization.

**Validity of physical model**: Replacing atmospheric scattering with Retinex decomposition in PGB reduces PSNR from 23.5 dB to 22.1 dB and mAP from 72.4% to 70.2%, confirming the superiority of the atmospheric scattering model for low-light scenes.

These results indicate that the introduction of physical priors indeed enhances the generalization ability and robustness of the model, enabling the network to better handle scenarios outside the distribution of training data.

### 5.7. Generalization Performance Analysis of Large-Scale Datasets

To evaluate the performance of PGT-Net on large-scale datasets, this article conducted comparative experiments on the complete BDD100K test set, and the results are shown in Table 7.

The results indicate that PGT-Net still achieved the best performance on the large-scale and diverse BDD100K dataset, mAP@0.5 reached 63.2%, an increase of 4.7% compared to the suboptimal method. This confirms the good scalability and robustness of PGT-Net in complex scenarios.

### 5.8. Limitation Analysis

Despite the excellent performance achieved by PGT-Net, several limitations remain that suggest directions for future research:

1. Computational complexity and edge deployment. Compared to lightweight enhancement methods (e.g., Zero-DCE), PGT-Net has higher parameter (19.2 M) and computational (68.5 G FLOPs) costs. Although it meets real-time requirements on RTX 3090 (31.3 FPS), deployment on resource-constrained edge devices (e.g., Jetson AGX Orin) yields only 14.7 FPS (FP16). Future work will explore model pruning, knowledge distillation [56], and INT8 quantization to reduce inference latency while preserving performance.
2. Extreme low-light conditions. Under illumination below 0.1 lux, single-frame RGB images contain insufficient information for reliable enhancement. PGT-Net’s restoration capability is limited, with PSNR dropping to 18.2 dB and mAP to 58.3% when SNR < 10 dB. Future work can integrate multi-frame fusion, infrared thermal imaging, or multispectral data to provide complementary information.
3. Dynamic scenes and motion blur. For fast-moving objects (e.g., vehicles > 60 km/h), the enhancement process may introduce or amplify motion blur, reducing detection recall by approximately 8%. Incorporating temporal information through 3D convolutions or video Transformers could enable joint video enhancement and detection.
4. Sensitivity to physical parameter estimation. The model’s detection performance degrades by >8% when Gaussian noise (σ > 20%) is added to the estimated illumination map A. Introducing uncertainty estimation mechanisms could dynamically adjust the weight of physical priors when predictions are unreliable.
5. Cross-dataset generalization. Models trained on ExDark show a 4.2 dB PSNR drop and 7.1% mAP drop when tested on MIT-Adobe FiveK. Future work could explore domain adaptation or unsupervised domain generalization techniques.
6. Limited modeling of complex weather conditions. The current physical guidance branch is based solely on the atmospheric scattering model, which cannot fully account for degradation from rain, snow, or fog. Developing a unified “all-weather” physical model that integrates multiple degradation mechanisms is a promising direction.

### 5.9. Summary of This Chapter

This chapter comprehensively evaluated PGT-Net through ablation studies, comparison with state-of-the-art methods, and generalization tests. Results demonstrate that PGT-Net achieves superior performance in both image enhancement quality and object detection accuracy, while maintaining a good balance between complexity and speed. The effectiveness of physical guidance and dual-branch design is validated.

## 6. Conclusions

### 6.1. Work Summary

This article proposes a novel physics-guided Transformer–CNN Hybrid Network PGT-Net for end-to-end joint optimization of low-light enhancement and object detection, aimed at addressing the visual perception challenges of intelligent transportation systems in low-light environments. The main contributions and advantages of this article are as follows:

Innovative physics data fusion paradigm: This paper propose a learnable physics-guided branch that integrates atmospheric scattering models into the network in a deep and learnable manner, significantly enhancing the model’s physical interpretability and generalization ability under unknown lighting conditions. The experiment showed that the introduction of this branch increased the detection mAP by 2.6% (see Table 3).

Efficient local global feature complementarity architecture: By designing a parallel CNN and Transformer dual-branch enhanced backbone and utilizing Physical Fusion Modules to achieve deep feature interaction, the trade-off between local detail restoration and global illumination correction has been successfully solved. The ablation experiment proved that the dual-branch design has a synergistic effect of “1 + 1 > 2”.

Task-driven end-to-end joint optimization: Seamlessly integrate the enhancement module with the detection module, and use multi-task loss joint training to directly serve the detection task in the enhancement process. On the BDD100K night dataset, PGT-Net will detect mAP@0.5. The significant increase from 58.7% without enhancement to 72.4% proves the effectiveness of task-driven optimization.

Crucially, our analysis of the interaction between enhancement and detection modules (Section 5.5) reveals that the performance gain stems not only from improved image quality (+11.4% mAP) but also from task-oriented feature learning enabled by joint optimization (+2.3% mAP). Mutual information analysis confirms that end-to-end training aligns the feature representations of the enhancement module with the detector’s downstream requirements, a key insight that distinguishes our work from conventional two-stage approaches.

Superior comprehensive performance: Experiments on multiple authoritative datasets have shown that PGT-Net achieves the current optimal level in image enhancement quality (PSNR/SSIM) and object detection accuracy (mAP), while maintaining a real-time inference speed of 31.3 FPS, achieving a good balance between accuracy and efficiency.

### 6.2. Future Work Outlook

Although PGT-Net has achieved excellent performance, there are still the following limitations that deserve further research in the future:

Computational complexity: Compared to pure lightweight enhancement methods such as Zero-DCE, PGT-Net still has higher parameter and computational complexity (19.2 M parameters, 68.5 G FLOPs). Future work can explore technologies such as model pruning and knowledge distillation to better deploy on resource-constrained edge devices.

Extreme low-light scenario: In extremely dark environments with illumination below 0.1 lux, single-frame RGB image information is severely missing, and the recovery ability of PGT-Net is limited. In the future, fusion perception can be achieved by combining multi-frame information, infrared thermal imaging, or multispectral images.

Dynamic scene processing: For high-speed moving targets (such as fast-moving vehicles), the enhancement process may introduce or amplify motion blur. Future work can introduce temporal information (such as 3D convolution or video Transformer) to construct a joint model for video enhancement and detection.

More complex physical models: Currently, physical guidance based on atmospheric scattering models has limited modeling capabilities for complex weather conditions such as fog, rain, and snow. In the future, it is possible to explore the construction of a unified “all weather” physical guidance model to adapt to a wider range of harsh environments.

### 6.3. Final Conclusions

The safe operation of autonomous driving relies heavily on reliable environmental perception across all weather conditions and various scenarios. Visual perception under low-light conditions stands as one of the most critical technical bottlenecks. The PGT-Net proposed in this paper successfully achieves significant performance improvements in low-light traffic image enhancement and object detection tasks by creatively integrating prior knowledge from physical models, the local detail extraction capabilities of CNNs, and the global context modeling abilities of Transformers, and implementing end-to-end enhancement–detection joint optimization.

This study not only provides a high-performance and interpretable solution for low-illumination visual perception, but also offers valuable exploration and practice in the deep integration of physical models and deep learning, as well as the application of multi-task joint learning in the field of computer vision. Experimental results demonstrate that PGT-Net achieves state-of-the-art performance on multiple benchmark datasets, while maintaining real-time processing capabilities, highlighting its strong practical application value.

With the continuous development and popularization of autonomous driving technology, the demand for robust perception in harsh environments will become increasingly urgent. We believe that this research will make a positive contribution to promoting the development of autonomous driving and intelligent transportation systems in challenging environments such as low illumination, and provide new ideas and directions for research in related fields.

## Figures and Tables

**Table 1:** Comparison of main low-light image enhancement methods.

Method Category	Representative Method	Advantage	Disadvantage
Traditional	Histogram equalization, Retinex, dehazing models, Retinex	Strong physical interpretability; no training data required	Manual parameter tuning; prone to halo, over-enhancement, noise; poor robustness
Supervised CNN-based	RetinexNet, KinDRetinexNet, KinD	Significant enhancement; learns complex mappings	Relies on high-quality paired data; limited receptive field; poor generalization
Unsupervised/Self-supervised	Zero-DCE, EnlightenGANZero-DCE, EnlightenGAN	No paired data needed; flexible training	Unstable enhancement; unnatural textures; limited handling of extreme lighting
Transformer-based	Uformer, RestormerUformer, Restormer	Models global dependencies; maintains global consistency	High computational cost; weaker local detail recovery; large parameter count

**Table 2:** Global Transformer Branch (GTB) configuration.

Stage	Output Size	Swin Transformer Block Type	Number of Channels	Number of Heads	Window Size
Patch Embed	H/4 × W/4	-	C = 96	-	-
Stage 1	H/4 × W/4	Regular window self-attention × 2	96	3	7 × 7
Stage 2	H/8 × W/8	Regular window self-attention × 2	192	6	7 × 7
Stage 3	H/16 × W/16	Regular window self-attention × 6	384	12	7 × 7

Note: The window size M is set to 7 for all stages, consistent with the Swin–Tiny configuration [31]. The shift size is M/2 = 3 for shifted window attention.

**Table 3:** Ablation experiment results of PGT-Net on ExDark test set.

Model Variant	PGB	GTB	PFM	End-to-End	mAP@0.5 (%)	PSNR (dB)	SSIM	Number of Parameters (M)
A (Baseline)					65.2	18.7	0.683	8.4
B	√				67.8 (+2.6)	20.1 (+1.4)	0.721 (+0.038)	9.1
C		√			69.1 (+3.9)	19.8 (+1.1)	0.708 (+0.025)	12.3
D	√	√			71.5 (+6.3)	21.3 (+2.6)	0.765 (+0.082)	13.0
E	√	√	√		73.9 (+8.7)	22.8 (+4.1)	0.812 (+0.129)	16.8
**F** (PGT-Net)	√	√	√	√	76.4 (+11.2)	23.5 (+4.8)	0.837 (+0.154)	19.2
G (w/o $L_{phy}$)	√	√	√	√	74.8(+9.6)	22.9	0.821(+0.138)	19.1

**Table 4:** Comparison of image enhancement quality of different methods on ExDark and SID test sets.

Method	ExDark			SID		
	PSNR (dB)	SSIM	LPIPS	PSNR (dB)	SSIM	LPIPS
LIME	17.3	0.62	0.351	16.8	0.59	0.398
RetinexNet	19.1	0.71	0.285	18.5	0.69	0.321
KinD	21.5	0.83	0.192	20.9	0.81	0.210
Zero-DCE	20.8	0.79	0.205	20.2	0.77	0.228
EnlightenGAN	20.1	0.78	0.198	19.7	0.76	0.215
Uformer	22.1	0.85	0.178	21.4	0.83	0.195
PGT-Net (Ours)	23.5	0.87	0.180	22.7	0.85	0.188

**Table 5:** Comparison of model complexity (input size: 512 × 512).

Method	Number of Parameters (M)	FLOPs (G)	Inference Time (ms)	FPS	Memory (GB)
IME	-	-	120	8.3	-
RetinexNet	0.85	45.2	25	40.0	1.2
KinD	8.13	187.5	65	15.4	2.8
Zero-DCE	0.079	4.1	8	125.0	0.8
Uformer	50.6	125.3	45	22.2	3.5
PGT-Net	19.2	68.5	32	31.3	2.1

**Table 6:** Comparison of object detection performance on the BDD100K-night test set.

Enhancement Method	mAP@ 0.5 (%)	mAP@0.5: 0.95 (%)	Pedestrian AP (%)	Vehicle AP (%)	FPS
No enhancement (original low-light image)	58.7	32.1	45.2	72.3	120
LIME	60.2 (+1.5)	33.5 (+1.4)	47.1	73.4	115
RetinexNet	62.5 (+3.8)	35.8 (+3.7)	49.8	75.3	112
KinD	66.3 (+7.6)	38.9 (+6.8)	53.4	79.2	108
KinD (two-stage, independent training)	65.8(+7.1)	38.5(+6.4)	52.9	78.7	105
Zero-DCE	64.1 (+5.4)	37.2 (+5.1)	51.2	77.0	118
Uformer	67.1 (+8.4)	39.5 (+7.4)	54.1	80.1	95
JLOD (Joint)	68.5 (+9.8)	40.1 (+8.0)	55.3	81.7	98
PGT-Net + YOLOv5-s	71.6(+12.9)	42.9(+10.8)	59.1	84.3	108
PGT-Net + YOLOv7-tiny	72.4(+13.7)	43.6(+11.5)	59.8	85.0	102
PGT-Net + YOLOv8-nano	71.9(+13.2)	43.1(+11.0)	59.4	84.7	115
PGT-Net + YOLOv21	71.8(+13.1)	43.0(+10.9)	59.2	84.8	128

**Table 7:** Comparison of object detection performance on the complete BDD100K test set.

Method	mAP@0.5 (%)	mAP@0.5:0.95 (%)
No enhancement (original low-light image)	51.3	28.5
KinD + YOLOv7-tiny	57.8	32.1
Zero-DCE + YOLOv7-tiny	55.6	30.9
Uformer +YOLOv7-tiny	58.5	33.0
**PGT-Net (Ours)**	**63.2**	**36.7**

## Data Availability

The original contributions presented in this study are included in the article material. Further inquiries can be directed to the corresponding author.
